# In Vitro Analysis of Probiotic Properties Related to the Adaptation of *Levilactobacillus brevis* to Intestinal Microenvironment and Involvement of S-Layer Proteins

**DOI:** 10.3390/ijms26062425

**Published:** 2025-03-07

**Authors:** Nina Čuljak, Krešo Bendelja, Andreja Leboš Pavunc, Katarina Butorac, Martina Banić, Ana Savić Mlakar, Željko Cvetić, Jana Hrsan, Jasna Novak, Jagoda Šušković, Blaženka Kos

**Affiliations:** 1 Department of Biochemical Engineering, Faculty of Food Technology and Biotechnology, University of Zagreb, Pierottijeva 6, 10000 Zagreb, Croatia; nina.culjak@pbf.unizg.hr (N.Č.); andreja.lebos.pavunc@pbf.unizg.hr (A.L.P.); katarina.butorac@pbf.unizg.hr (K.B.); martina.banic@pbf.unizg.hr (M.B.); jana.hrsan@gmail.com (J.H.); jsusko@pbf.hr (J.Š.); blazenka.kos@pbf.unizg.hr (B.K.); 2 Center for Research and Knowledge Transfer in Biotechnology, University of Zagreb, Rockefellerova 10, 10000 Zagreb, Croatia; kreso.bendelja@unizg.hr (K.B.); ana.savic@unizg.hr (A.S.M.); zeljko.cvetic@unizg.hr (Ž.C.)

**Keywords:** S-layer proteins, *Levilactobacillus brevis*, immunomodulation, probiotic, adhesion, microbiota

## Abstract

Although rare, the ability to produce surface S-layer proteins is beneficially associated with particular *Lactobacillus* strains being investigated as probiotics. Therefore, this work aimed to study specific probiotic functionalities of selected *Levilactobacillus brevis* strains MB1, MB2, MB13 and MB20, isolated from human milk microbiota, and to assess the contribution of S-proteins. Firstly, Rapid Annotation using Subsystem Technology revealed that cell wall-related genes were abundant in analysed *L. brevis* genomes. Furthermore, the results demonstrated that S-proteins mediate aggregation capacity and competitive exclusion of selected pathogens by *L. brevis* strains. The improvement of Caco-2 epithelial monolayer barrier function was demonstrated by the increase in JAM-A and occludin expressions when *L. brevis* strains or S-proteins were added, with the effect being most pronounced after treatment with MB2 and S-proteins of MB1. *L. brevis* strains, especially MB20, exerted the potential to adhere to recombinant human ZG16. Strain MB2 and MB20-S-proteins improved the barrier function of HT29 epithelial monolayer, as evidenced by increased ZG16 expression. Analysed *L. brevis* strains and S-proteins differentially affected the protein expression of IL-1β, IL-6 and IL-8, and IL-10 cytokines. The most prominent effect was observed by S-proteins of MB20, since IL-1β production was decreased while IL-10 production was significantly increased.

## 1. Introduction

Probiotic strains, including those belonging to the lactic acid bacteria (LAB), have been described as having diverse functions, such as the exclusion of pathogens through competition for nutrients and receptors for their binding, production of antimicrobial substances that inhibit their growth, promotion of epithelial barrier function by increasing the production of mucins and the expression of tight junction (TJ) proteins, preventing the translocation of pathogens from the gut into the blood, and even by regulating the neuro-immune axis [1,2]. The in vivo efficacy of probiotics is determined by their ability to adapt to the specific conditions of the digestive tract, but also by their interactions with resident bacteria and host cells. Specific mechanisms and clinical outcomes of probiotic strains are mediated by probiotic cell components or cell debris and, more recently, there is also evidence that non-living cells can also act as health promoters with enhanced bioactivity, which has contributed to the development of the concept of postbiotics [3]. The term postbiotics refers to soluble factors, such as metabolites or by-products, that are secreted by live bacteria or released after bacterial lysis and can have a beneficial effect on the consumer. These ingredients have production advantages over probiotics as cell viability is not required, which simplifies the manufacturing process and application [4]. Most in vitro and in vivo studies have focused on postbiotics derived from various LAB strains [5,6]. *Lactobacillus* S-layer proteins are hypothesised to have postbiotic characteristics and can be considered as probiotic effector molecules. S-layer proteins are the first to interact with the microenvironment and contribute to the probiotic effect of the strain that produces them [7]. Their protective role in stressful conditions, in mediating interactions between mucus and epithelial cells, as well as their immunomodulatory effect have been demonstrated [8,9,10,11].

Our research group has previously isolated *Lactobacillus* strains, producing potentially therapeutic biomolecules, including the S-proteins [12,13]. The role of S-proteins for *Levilactobacillus brevis* strains MB1, MB2, MB13 and MB20, isolated from mother’s milk, in terms of better survival under simulated gastrointestinal tract (GIT) conditions and better adhesion to Caco-2 cells was confirmed [14]. The aim of this work was to investigate the role of S-proteins and *Lactobacillus* strains that produce them, by performing in vitro analysis of specific properties related to the adaptation and effect of probiotics in the intestinal milieu, more specifically by targeted characterization of adhesion capacities, ability to impact intestinal barrier function, and assessment of stimulation of specific cytokines production.

## 2. Results and Discussion

### 2.1. Genome Features of L. brevis Strains

Accurate taxonomic identification of probiotic strains is essential since their safety and health effects are strain dependent. Therefore, the genomes of S-protein-producing *L. brevis* strains MB1, MB2, MB13 and MB20, isolated from human milk microbiota, were sequenced and deposited in the NCBI database (BioProject PRJNA388578) under accession numbers SAMN22155538, SAMN22155539, SAMN22155540 and SAMN221555341, respectively [13]. In addition, the annotation, distribution and categorisation of the genes of the analysed strains was performed using the RAST server, on which all data on the sequenced genomes of the *L. brevis* strains MB1, MB2, MB13 and MB20 are stored and available (Figure S1). Genome annotation using the RAST server revealed that genes related to the cell wall and capsule represent one of the most abundant gene groups of *L. brevis* strains MB1, MB2, MB13 and MB20, which is in line with expectations, as S-proteins account for about 10–20% of the total cellular proteins of the producer bacteria.

Comparison of the genome data of *L. brevis* strains MB1, MB2, MB13 and MB20 (Table S1), also obtained using the RAST server, shows that there are no large jumps in the highlighted values between the individual *L. brevis* strains MB1, MB2, MB13 and MB20 and that the genome features obtained are consistent with previously published genome sequences of *L. brevis* strains isolated from different microhabitats [8,10,15]. The results obtained show a high degree of overlap between the studied genomes, which was to be expected as they belong to the same species and produce S-proteins.

### 2.2. Role of S-proteins in the Properties Important for Intestinal Adaptation of L. brevis Strains

Čuljak et al. [14] previously demonstrated the role of S-proteins of *L. brevis* strains with regard to better survival under simulated GIT conditions and improved adhesion to Caco-2 cells. In this paper, following previous experiments, autoaggregation and coaggregation assays, as well as competitive exclusion of potentially pathogenic bacteria *E. coli* 3014 and *S.* Typhimurium FP1 were performed. Autoaggregation, i.e., the cluster of bacteria of the same strain, is thought to be the first step in adhesion to epithelial cells and mucosal surfaces, allowing bacteria to form a barrier by colonising the GIT and thus preventing the adhesion of undesirable bacteria [16]. Similarly, coaggregation, i.e., the aggregation of two different bacterial strains, of probiotic bacteria with pathogens can also prevent pathogens from colonising the GIT [17].

The percentage of autoaggregation was lower in all *L. brevis* strains after removal of the S-layer, with S-proteins of *L. brevis* strains MB1 and MB20 being the most pronounced (*p* < 0.001). Our results confirm that S-layer proteins, as the outermost barrier surrounding the cell surface, play an important role in the autoaggregation process. These results are in agreement with those of Alp et al. [18], who also found a role of S-proteins in the autoaggregation of *L. casei* DA4, *L. coryniformis* DA263, *L. plantarum* DA100 and DA140, as evidenced by a reduced aggregation ability after removal of the S-layer by LiCl treatment.

The results related to the characterisation of the coaggregation of *L. brevis* strains indicated a role of S-proteins when coaggregating with *S.* Typhimurium FP1, since the percentage of coaggregation was significantly lower after S-layer removal, with the greatest difference observed for strain MB13 (*p* < 0.001) (Table 1). Regarding coaggregation with *E. coli* 3014, only strains MB1 and MB2 showed a statistically significant increase in coaggregation ability when S-proteins were present compared to GHCl-treated strains (*p* < 0.05), while there was no difference in strains MB13 and MB20 (Table 2). These results indicate that S-proteins do not play a crucial role in aggregation mechanisms in all strains expressing them, but that other cell wall components may play a more important role. Our results are consistent with the findings of Alp et al. [18], who demonstrated a role of S-proteins of strains *L. casei* DA4, *L. coryniformis* DA263 and *L. plantarum* DA140 in coaggregation with Salmonella enterica serovar Enteritidis, with the exception of *L. plantarum* DA100.

**Figure 1:**
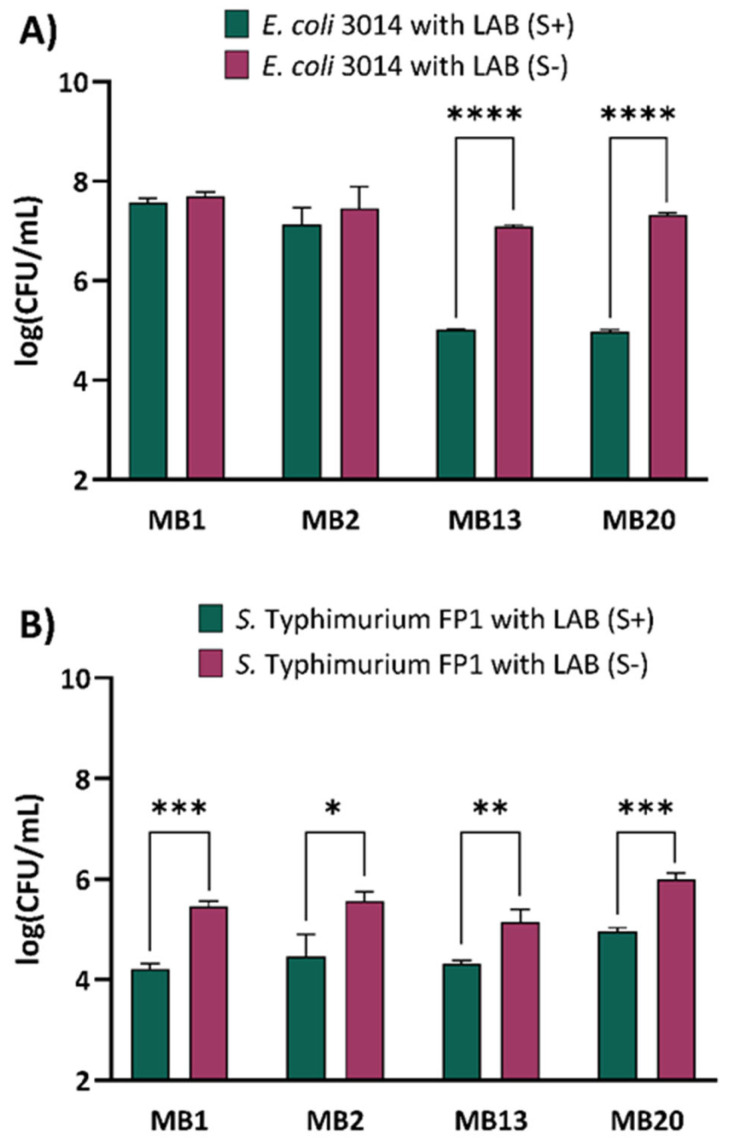
Competitive exclusion of potentially pathogenic bacteria (**A**) *Escherichia coli* 3014 and (**B**) *S. serovar* Typhimurium FP1 after pre-incubation of Caco-2 cells with *Levilactobacillus brevis* MB1, MB2, MB13 and M20 strains, with (S+) or without (S−) S-layer. The error bars represent the standard deviations of the mean values of the results of three replicates. * Statistically significantly different (* *p* < 0.05, ** *p* < 0.01, *** *p* < 0.001, **** *p* < 0.0001), according to Tukey’s honestly significant difference (HSD) test.

Competitive exclusion is defined as a process in which one bacterial species competes with another bacterial species for adhesion to receptors in the GIT, as manifested by the ability to bind to the surface of the host’s mucosa, secretion of various metabolites and competition for available nutrients [19]. Probiotics with the ability to adhere to the intestinal epithelium are prone to preventing colonisation with intestinal pathogens. *Lactobacillus rhamnosus* GG has been shown to be effective against infection with vancomycin-resistant enterococci (VRE) as it excludes *Enterococus faecium*, which can easily acquire new resistance to antibiotics and is a successful coloniser of the human intestinal tract [20]. Ayala et al. [21] also demonstrated that certain LAB strains have the ability to competitively exclude *Salmonella* Montevideo, *Escherichia coli* O157:H7 and *Listeria monocytogenes* N1-002. Zhang et al. [22] point the role of S-layer protein extracts in preventing the adhesion of pathogens and thus intestinal colonisation. The competitive exclusion of *E. coli* 3014 and *S.* Typhimurium FP1 and the involvement of S-layer were analysed on Caco-2 cells with the ability to spontaneously differentiate into a monolayer having similar properties as small intestine enterocytes [22]. The results showed that S-proteins of *L. brevis* strains MB13 and MB20 contributed to the competitive exclusion of *E. coli* 3014 (Figure 1A), as a statistically significant decrease in log(CFU/mL) values was observed when S-proteins were present on the cell surface (*p* < 0.0001). The analysed strains successfully excluded the adhesion of *S.* Typhimurium FP1 as a significant increase in log(CFU/mL) values was observed after removal of the S-layer compared to the results achieved followed by S-protein extraction (Figure 1B). A particularly pronounced effect in reducing the ability of the competitive exclusion was observed in *L. brevis* strains MB1 and MB20 as there was a highly statistically significant difference between the intact and S-layer-depleted strain (*p* < 0.001), suggesting a mediating role of S-proteins. This suggests that *L. brevis* S-proteins have a potential role in the competitive exclusion of pathogenic bacteria and possibly affinity for the Caco-2 cell line. The results support the hypothesis that S-proteins mediate the interaction between producer cells and intestinal epithelium [14]. Noteworthy, the results demonstrated that four *L. brevis* strains, with or without S-layer, reduced the cell adhesion of *E. coli* 3014 and *S.* Typhimurium FP1 to Caco-2 cells, as evident from the significantly higher cell counts of *E. coli* 3014 and *S.* Typhimurium FP1, which reached levels of 8.45 ± 0.18 and 6.43 ± 0.25 log(CFU/mL) in control experiments, respectively.

### 2.3. Capacity of L. brevis Strains and Their S-proteins to Improve Epithelial Barrier Function In Vitro

The intestinal epithelium maintains a balance between the selective active and passive transport of molecules, forming a physical barrier that protects against the invasion of various pathogens [23,24]. The intercellular TJ proteins regulate the passive transport of luminal fluid and molecules between epithelial cells. TJs are the most apical intercellular junctions of epithelial cells formed by the transmembrane proteins occludins, claudins and junctional adhesion molecules (JAMs), as well as peripheral membrane proteins such as zonula occludens (ZO) [25]. The altered expression can lead to a loss of intestinal barrier integrity, which allows uncontrolled transfer of the intestinal microbiota and intestinal content submucosally, following the activation of the inflammatory process [26].

The secretory intestinal proteins like mucin MUC2 also represent an important physical component of the intestinal protective barrier by covering the intestinal epithelial cells and forming an intestinal mucosal layer that improves food absorption, provides attachment sites for symbiotic intestinal bacteria and limits pathogen colonisation [27]. Another important component of the extracellular mucus layer is the protein ZG16 present throughout intestine [28], which crosslinks a polymer network of mucins and other secreted mucus glycoproteins. Primary ZG16 is responsible for intestinal mucus stratification and the agglomeration of Gram-positive microbiota in the distal mucus layer, preventing them from reaching the intestinal epithelial cells [29]. It shows an anti-inflammatory and anti-tumour activity, preventing bacteria from binding to the colonic epithelium [28]. Research has shown that probiotics increase the expression of genes encoding the aforementioned TJ proteins and regulate the expression of mucin, thereby influencing the formation of the mucus layer and maintaining the integrity of the intestinal epithelial barrier [27,30,31]. In addition to the intact LAB cells, some of their metabolites also show an effect on the maintenance of the intestinal barrier [27].

**Figure 2:**
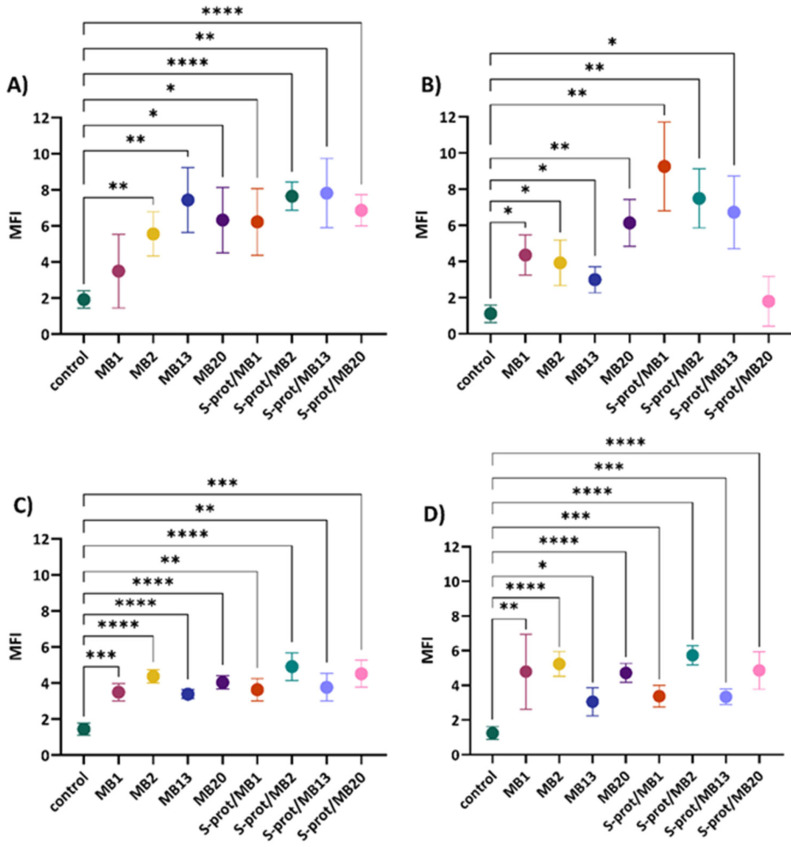
JAM-A expression in Caco-2 cells after establishing a monolayer and cultivation in (**A**) medium, (**B**) medium supplemented with LPS, (**C**) medium supplemented with TNF-α and (**D**) medium supplemented with LPS and TNF-α, after exposure to *L. brevis* MB1, MB2, MB13 and MB20 strains or their S-proteins. MFI—mean fluorescence intensity; control—protein expression when Caco-2 cells were not exposed to *L. brevis*/S-proteins. The error bars represent the standard deviations of the mean values of the results of three replicates. * Statistically significantly different (* *p* < 0.05, ** *p* < 0.01, *** *p* < 0.001, **** *p* < 0.0001) according to Tukey’s honestly significant difference (HSD) test.

**Figure 3:**
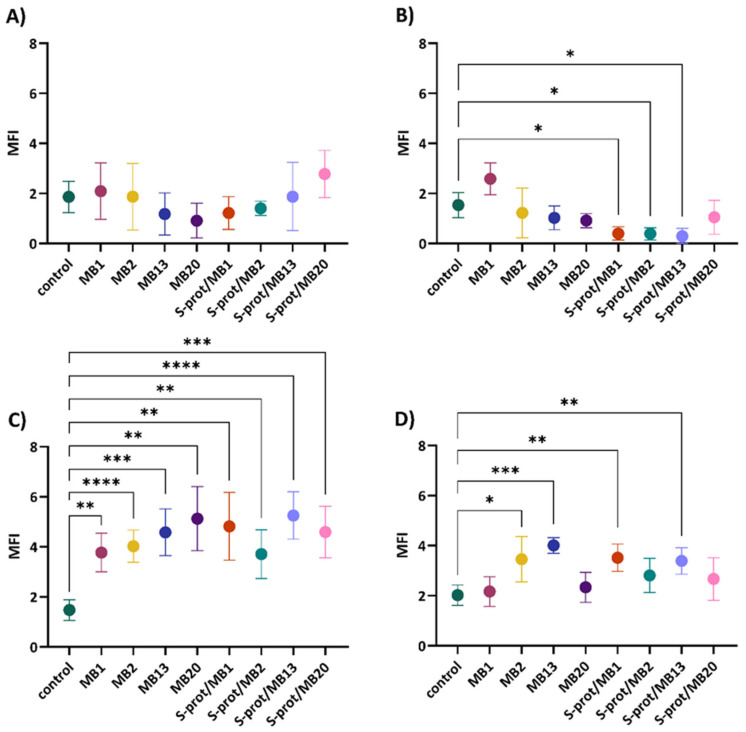
Occludin expression in Caco-2 cells after establishing a monolayer and cultivation in (**A**) medium, (**B**) medium supplemented with LPS, (**C**) medium supplemented with TNF-α and (**D**) medium supplemented with LPS and TNF-α, after exposure to *Levilactobacillus brevis* MB1, MB2, MB13 and MB20 strains or their S-proteins. MFI—mean fluorescence intensity; control—protein expression when Caco-2 cells were not exposed to *L. brevis*/S-proteins. The error bars represent the standard deviations of the mean values of the results of three replicates. * Statistically significantly different (* *p* < 0.05, ** *p* < 0.01, *** *p* < 0.001, **** *p* < 0.0001) according to Tukey’s honestly significant difference (HSD) test.

**Figure 4:**
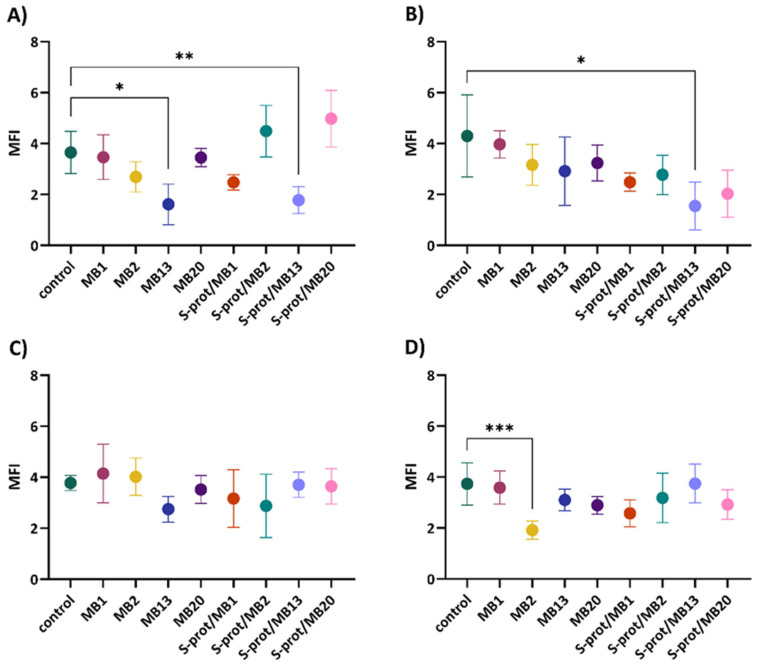
ZO-1 expression in Caco-2 cells after establishing a monolayer and cultivation in (**A**) medium, (**B**) medium supplemented with LPS, (**C**) medium supplemented with TNF-α and (**D**) medium supplemented with LPS and TNF-α, after exposure to *Levilactobacillus brevis* MB1, MB2, MB13 and MB20 strains or their S-proteins. MFI—mean fluorescence intensity; control—protein expression when Caco-2 cells were not exposed to *L. brevis*/S-proteins. The error bars represent the standard deviations of the mean values of the results of three replicates. * Statistically significantly different (* *p* < 0.05, ** *p* < 0.01, *** *p* < 0.001), according to Tukey’s honestly significant difference (HSD) test.

**Figure 5:**
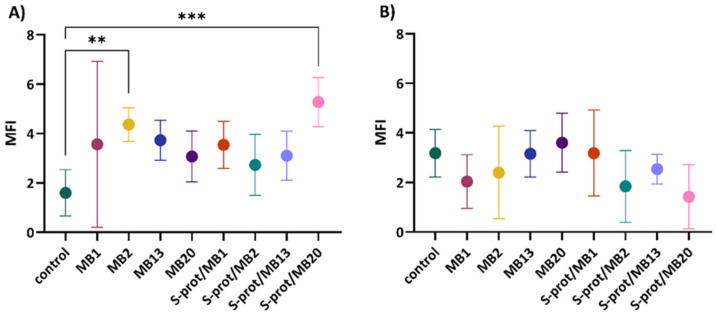
ZG16 expression in HT29MTX+5FU cells after establishing a monolayer and cultivation in (**A**) medium and in (**B**) medium supplemented with TNF-α, and subsequent exposure of LAB *Levilactobacillus brevis* MB1, MB2, MB13 and MB20 strains or their S-proteins. MFI—mean fluorescence intensity; control—protein expression when HT29MTX+5FU cells were not exposed to *L. brevis*/S-proteins. The error bars represent the standard deviations of the mean values of the results of three replicates. * Statistically significantly different (** *p* < 0.01, *** *p* < 0.001) according to Tukey’s honestly significant difference (HSD) test.

Therefore, the role of *L. brevis* strains MB1, MB2, MB13 and MB20 and their S-proteins in improving the epithelial monolayer barrier function of human cell cultures was investigated by monitoring the expression of JAM-A, occludin and ZO-1, and ZG16 by fluorescence microscopy (Figure S2). In an experimental setup, the expression of individual TJ proteins after treatment with *L. brevis* strains and derived S-proteins was monitored under four different conditions, simulating some different possible scenarios in the intestine. These included conditioning with a medium that represents the homeostatic specific pathogen-free (SPF) state in the gut (as in Figure 2, Figure 3, Figure 4 and Figure 5); a medium preconditioned with LPS mimicking the presence of Gram-negative pathogens such as *E. coli* (Bs in Figure 2, Figure 3, Figure 4 and Figure 5); a medium pretreated with TNF-representing inflammatory conditions (Cs in Figure 2, Figure 3, Figure 4 and Figure 5); and lastly, a medium exposed to TNF and LPS which should simulate the combination of inflammatory conditions and the presence of pathogenic *E. coli* (Ds in the Figure 2, Figure 3, Figure 4 and Figure 5). According to the results, a statistically significant increase in JAM-A expression was observed in cells grown in the medium (Figure 2A) after exposure to MB2 (*p* < 0.01), MB13 (*p* < 0.01) and MB20 (*p* < 0.05) strains and S-proteins of MB1 (*p* < 0.05), MB2 (*p* < 0.0001), MB13 (*p* < 0.01) and MB20 (*p* < 0.0001) strains. When the cells were treated with LPS (Figure 2B), there was a statistically significant increase in JAM-A expression after exposure to all *L. brevis* strains tested (*p* < 0.05 for MB1, MB2 and MB13, *p* < 0.01 for MB20) and S-proteins of strains MB1 (*p* < 0.01), MB2 (*p* < 0.01) and MB13 (*p* < 0.05). Furthermore, since the addition of TNF and LPS to the medium emulates a proinflammatory environment in the intestine, we also studied whether treatment of analysed *L. brevis* strains and derived S-proteins in such conditions would exert a protective activity in preserving the Caco-2 cells’ monolayer. Simultaneous addition of S-proteins or *L. brevis* strains together with TNF-α (Figure 2C) resulted in a statistically significant increase in JAM-A expression, with strain MB2 and its S-proteins being the most pronounced (*p* < 0.0001). Similarly, the simultaneous addition of S-proteins or intact producer cells together with TNF-α and LPS (Figure 2D) resulted in a statistically significant increase in JAM-A expression, with strains MB2 and MB20 and their S-proteins standing out the most (*p* < 0.0001). The results of increased expression of JAM-A suggest a role of *L. brevis* strains and their S-proteins in maintaining the TJ integrity in homeostasis and inflammation. These results are consistent with the findings of Yin et al. [32] on *L. plantarum* CGMCC 1258, which expresses surface layer and which have shown that its integral domain micro integral membrane protein (MIMP) can restore damaged TJ function by increasing the expression of JAM-1, occludin and claudin-1.

Further analysis demonstrated that the addition of *L. brevis*/S-proteins had no significant effect on the expression of occludin in the medium compared to the control (Figure 3A). However, when the cells were treated with LPS (Figure 3B), the addition of S-proteins of strains MB1, MB2 and MB13 significantly reduced occludin expression (*p* < 0.05). On the contrary, the addition of all *L. brevis*/S-proteins tested significantly increased the expression of occludin in the cells after the simultaneous addition of TNF-α (Figure 3C), with MB2 strain and S-proteins of MB13 strain being the most pronounced (*p* < 0.0001). Simultaneous treatment of cells with *L. brevis*/S-proteins together with TNF-α and LPS (Figure 3D) resulted in a statistically significant increase in occludin expression after the addition of MB2 (*p* < 0.05) and MB13 (*p* < 0.001) strains and S-proteins of MB1 (*p* < 0.01) and MB13 (*p* < 0.01) strains. The results of increased expression of occludin suggest a role of S-proteins and *L. brevis* in maintaining the integrity of TJ, which is consistent with the results of Bendinelli et al. [33] who demonstrated that S-layer proteins of lactobacilli positively modulate the expression of occludin.

Analysis of the results of the expression of ZO-1 in Caco-2 cells indicates a statistically significant decrease in the expression of this TJ protein after the addition of strain MB13 (*p* < 0.05) and its S-proteins (*p* < 0.01) (Figure 4A). Similarly, the addition of S-proteins of MB13 strain (*p* < 0.05) significantly reduced ZO-1 expression after LPS treatment (Figure 4B). The addition of *L. brevis*/S-proteins did not significantly alter the expression of ZO-1 after simultaneous treatment of cells with *L. brevis*/S-proteins and TNF-α (Figure 4C). On the other hand, simultaneous treatment of the cells with *L. brevis*/S-proteins together with TNF-α and LPS (Figure 4D) significantly reduced the expression of ZO-1 after treatment with MB2 strain (*p* < 0.001). Increased expression of ZO-1 is important for maintaining the intestinal barrier integrity [34]. However, in our study, ZO-1 expression shows a variable outcome depending on the treatment and exposure to the selected probiotic strain, which may be transient, and the balance could eventually be restored through different pathways.

Besides the densely O-glycosylated MUC2, proteomic analysis of colonic mucus identified a small lectin-like protein, ZG16, as a highly abundant protein [29]. Therefore, we further studied the ability of intact *L. brevis* cells or their S-proteins to influence ZG16 expression on the model of HT29 cells. Since ZG16 protein is a protein secreted by colon goblet cells, HT29 cells were used for the analysis of ZG16 expression as they show capability to differentiate into goblet-like cells when exposed to 10 µM MTX, up to 20%. During exposure of HT29 cells to MB2 strain (*p* < 0.01) and S-proteins expressed by MB20 strain (*p* < 0.001), statistically significant increased ZG16 expression was observed, while moderate expression was observed for the other analysed strains and their S-proteins (Figure 5A). Treatment of the cells with TNF-α was not found to result in a statistically significant change in ZG16 expression (Figure 5B). The results of increased expression of ZG16 under physiological conditions by MB2 and MB20 strains may be associated as having a role in maintaining intestinal homeostasis. It has been shown that decreased expression of ZG16 is associated with ulcerative colitis and colon cancer [35,36,37], both diseases characterised by chronic cytokine secretion and persistent intestinal inflammation. In our experimental setup, short-term treatment of HT29 cells with TNF-α alone increased ZG16 expression compared to untreated cells, similar to MUC2 upregulation in HM3 colon adenocarcinoma cells [38]. *L. brevis*/S-protein cotreatment with TNF-α showed no additive effect on ZG16 expression. However, TNF-α has been shown to play a pathological role in dextran sulphate sodium (DSS)-induced colitis where anti-TNF-α antibody treatment ameliorated disease. In the acute phase upon DSS onset, TNF-α upregulates MUC2 expression, whereas later in the disease course the loss of goblet cells and decreased MUC2 production are evident [39]. It would be worthwhile to investigate whether prolonged TNF-α treatment could influence ZG16 production in HT29 cells and whether *L. brevis*/S-proteins from the tested probiotic strains can modulate this pathological process.

The results indicate that *L. brevis* MB1, MB2, MB13 and MB20 or their S-proteins have a different effect on the expression of individual TJ proteins depending on the conditions under which Caco-2 cells were incubated. Among the strains tested, *L. brevis* MB2 stood out, although in the case of ZO-1 expression it caused a statistically significant decrease in Caco-2 cells incubated simultaneously with TNF-α and LPS. As for the S-proteins, those of strain MB20 caused the highest increase in the expression of JAM-A and occludin. Clearly, these data warrant further research to investigate the mechanism by which the S-layer bearing *Lactobacillus* strains exerts its effects on TJ proteins in the model of Caco-2 cells. The ability of the *Lactobacillus* strains analysed, particularly MB2, to improve Caco-2 barrier function, which may consequently prevent or even reverse the increase in epithelial permeability and thus cause pathogen exclusion, prompts further investigation that could, among others, also include further characterisation of polarized epithelial cell monolayers by measuring TEER to quantify the integrity of TJ between intestinal epithelial cells.

### 2.4. The Immunomodulatory Effect of L. brevis Strains and S-proteins In Vitro

**Figure 6:**
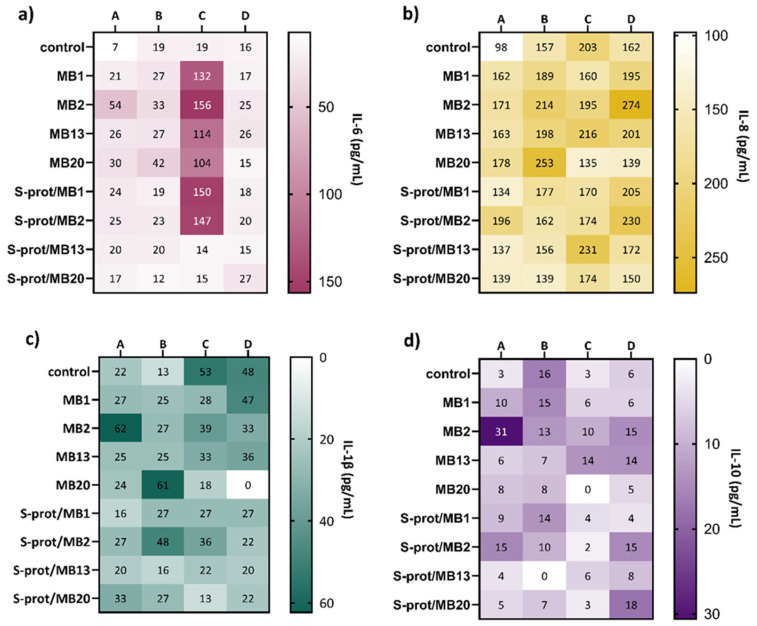
Effect of *Levilactobacillus brevis* MB1, MB2, MB13 and MB20 strains and their S-proteins on the production of IL-6 (**a**), IL-8 (**b**), IL-1β (**c**) and IL-10 (**d**) by Caco-2 cells (pg/mL) after the establishment of a monolayer and cultivation in (A) medium, (B) medium supplemented with LPS, (C) medium supplemented with TNF-α and (D) medium supplemented with LPS and TNF-α. control—expression when Caco-2 cells were not exposed to *L. brevis*/S-proteins.

Inflammatory bowel disease is associated with a dysfunction of the intestinal epithelial barrier causing a disruption of the selective permeability that allows the passage of intestinal contents, including the microbiota. This dysfunction is characterised by a reduced expression of proteins required for the functional assembly of the TJ. As a result, contact of subepithelial cells with bacteria leads to the initiation of inflammation associated with increased leukocyte infiltration and the production of pro-inflammatory cytokines IFN-γ, TNF-α and IL-1β [26]. Certain LAB strains have been shown to modulate the expression of pro-inflammatory cytokines, but the mechanisms involved are not yet fully understood [40]. Interestingly, *Lactobacillus* strains that produce S-proteins, which contribute to the survival of the producing cells under unfavourable environmental conditions, also display efficient adherence to the intestinal epithelial cells [14]. Along with adhesion, modulation of mucosal and systemic immune responses is an important mechanism in the action of probiotics. Therefore, investigating the specificity of immunomodulation induced by probiotic bacteria is of particular importance for their functionality in GIT. Previously, Johnson et al. [41] found that the S-layer proteins of *L. acidophilus* NCFM interact with DC-SIGN of dendritic cells, thereby triggering the differentiation of naïve T cells towards the Treg or Th2. Additionally, probiotic lactobacilli can induce pro-inflammatory cytokines such as interleukin ILβ-1, IL-6 and IL-8, as well as the anti-inflammatory cytokine IL-10 through interactions with intestinal epithelial cells as part of the host’s innate immune response recognised as protective immunity. These findings led us to investigate the potential of our S-layer-carrying *L. brevis* strains to stimulate cytokine secretion by Caco-2 cell line. In this context, the immunomodulatory effect of *L. brevis*/S-proteins was tested after mimicking inflammation by adding TNF-α and LPS (Figure 6). The levels of IL-6 increased after the addition of almost all *L. brevis*/S-proteins after incubation of Caco-2 cells only in the medium, with the largest increase with the MB2 strain (Figure 6a). When the Caco-2 cells were cultured in the medium with the addition of LPS, no significant change in IL-6 concentration was observed after the addition of *L. brevis*/S-proteins compared to the control. On the other hand, the simultaneous addition of *L. brevis*/S-proteins to TNF-α-treated Caco-2 cells increased IL-6 production after addition of all tested *L. brevis* strains and S-proteins of MB1 and MB2 strains. The IL-6 concentration in the control and after addition of *L. brevis*/S-proteins, after simultaneous incubation of Caco-2 cells in medium with TNF-α and LPS, is similar.

IL-8 production increased following the addition of almost all *L. brevis*/S-proteins after incubation of Caco-2 cells in medium only, with S-proteins of MB2 strain being the most prominent (Figure 6b). The production of IL-8 after addition of LPS did not change significantly after addition of *L. brevis*/S-proteins, except for strains MB2 and MB20, where the concentration increased. The simultaneous addition of almost all *L. brevis*/S-proteins tested and TNF-α resulted in a slight decrease in IL-8 production, except for MB13 strain and its S-proteins. After simultaneous treatment of cells with TNF-α and LPS, the IL-8 production increased after addition of MB2 strain and its S-proteins.

The production of IL-1β increased only after addition of the MB2 strain compared to the control when the Caco-2 cells were incubated in the medium (Figure 6c). The culture with *L. brevis*/S-proteins and LPS increased the production of IL-1β, especially with MB20 strain, whereas addition of TNF-α decreased IL-1β production compared to the control, with the largest decrease when MB20 strain and its S-proteins were added. A complete block of IL-1β production was observed following culturing the cells with MB20 strain and with TNF-α and LPS.

The results represent the mean values of duplicate samples pooled from five wells.

As can be observed from Figure 6d, the overall IL-10 production in different treatments is very low. The addition of *L. brevis*/S-proteins increased IL-10 production only after the treatment by MB2 strain. The culture with *L. brevis*/S-proteins and LPS did not alter the production of IL-10, with the exception of the S-proteins of MB13, which completely blocked IL-10 production. When TNF-α was added to Caco-2 cultured in the medium, MB13 increased IL-10 production compared to the control. IL-10 production increased after the addition of MB2 and MB13, as well as S-proteins of MB2 and MB20, when the cells were cultured in medium supplemented with LPS and TNF-α.

These experiments demonstrated the immunomodulatory effect of *L. brevis* strains and their S-proteins, mainly by modulating the production of pro-inflammatory cytokines. In a similar study, the application of *L. fermentum* CQPC04 significantly inhibited the production of pro-inflammatory cytokines IFN-γ, IL-1β, TNF-α, IL-6 and IL-12 and increased the release of the anti-inflammatory cytokine IL-10 in serum, which reduced colon damage [42]. The results obtained suggest the potential probiotic properties of these strains and their biomolecules, with S-proteins of strain *L. brevis* MB20, which reduced the expression of the IL-1β and increased the expression of the IL-10 standing out in particular.

### 2.5. Adhesion to ZG16 Protein

The intestinal epithelial cells are covered with a layer of mucus that prevents direct contact with the intestinal microbiota and also serves as a binding site for probiotic species [43]. One of the components of the mucus layer is the ZG16 protein, whose function is not yet fully understood but which is known to prevent bacterial invasion [36]. According to Bergström et al. [29], *Lactobacillus jensenii* peptidoglycan has been shown to bind ZG16. It is suggested that ZG16 aggregates bacteria and thereby cooperates with the inner colon mucus layer and limits bacterial translocation into the host [29,44].

**Figure 7:**
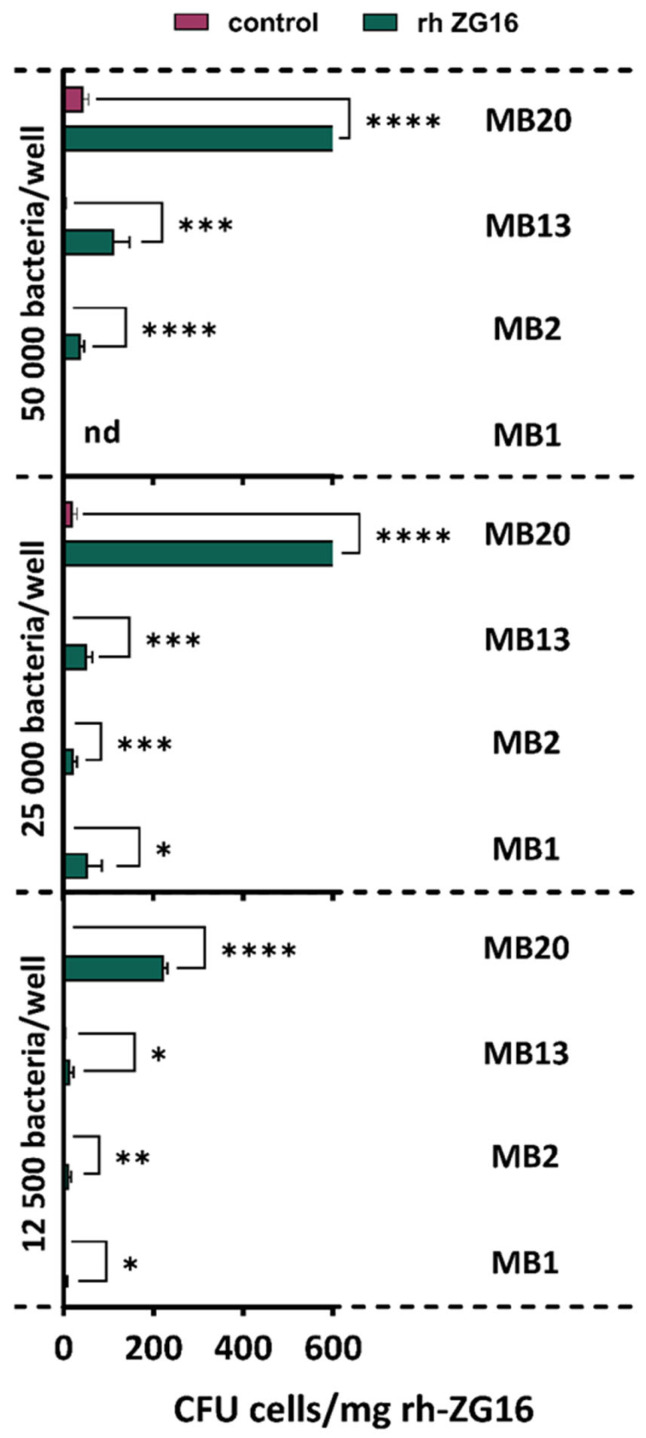
Adhesion of *L. brevis* strains MB1, MB2, MB13 and MB20 to recombinant human ZG16 protein (rh ZG16) and I-Block (control) when 50,000, 25,000 and 12,500 bacterial cells were added to the wells. nd—not done (concentration of the cells was too high in the stock solution). The error bars represent the standard deviations of the mean values of the results of three replicates. * Statistically significantly different (* *p* < 0.05, ** *p* < 0.01, *** *p* < 0.001, **** *p* < 0.0001).

The results of analysis of adhesion ability show that all *L. brevis* strains tested bind the hr ZG16 protein to some degree, with MB20 adhering most successfully, whereas MB1 has the weakest level of adhesion (Figure 7). Therefore, the possible role of S-proteins in the interaction with mucus components, but obviously adhesion properties are strain-specific. Phùng et al. [45] also investigated the ability of various probiotic strains to adhere to the mucus, of which *Lactiplantibacillus plantarum* and *Bifidobacterium longum* subsp. *infantis* showed a greater ability.

## 3. Materials and Methods

### 3.1. Bacterial Strains and Cell Lines

S-layer-producing *L. brevis* MB1, MB2, MB13 and MB20 were stored at −80 °C in de Man Rogosa Sharpe (MRS; Difco, Detroit, MI, USA) broth, while *E. coli* 3014 and *S. enterica* serovar Typhimurium FP1 were maintained in Brain Heart Infusion (BHI; Biolife, Milan, Italy) broth supplemented with 15% *v*/*v* glycerol (Alkaloid, Skopje, Macedonia). All strains used in this study are part of the Culture Collection of the Laboratory for Antibiotic, Enzyme, Probiotic and Starter Cultures Technology, University of Zagreb Faculty of Food Technology and Biotechnology. Prior to each experiment, all strains were subcultured twice MRS broth and incubated at 37 °C in microaerophilic conditions.

Caco-2 cells (American Type Culture Collection, ATCC HTB-37, Manassas, VA, USA) were stored in DMEM Ham’s F-12 medium containing L-glutamine (Capricorn Scientific, Ebsdorfergrund, Germany) with 10% FBS (Gibco, London, UK) and 10% dimethyl sulphoxide (DMSO; Kemika, Zagreb, Croatia) in liquid nitrogen. Caco-2 cells were routinely grown in 24-well plates until 80–90% confluent monolayers were reached and then gently rinsed three times with PBS (pH = 7.4). The HT-29 cell line was stored and prepared similarly to the Caco-2 cell line. Research activities were conducted in accordance with all regulatory quality and safety SOP requirements.

### 3.2. Functional Annotation of L. brevis Genomes

Previously isolated, sequenced and in NCBI deposited genomes of *L. brevis* MB1, MB2, MB13 and MB20 [13] were annotated using the RAST (Rapid Annotations using Subsystems Technology; http://rast.nmpdr.org/rast.cgi, accessed on 1 July 2024) server, by identifying protein-coding, rRNA and tRNA genes, assigning them a function, predicting which subsystems are present in the genome, and using the information obtained to construct the metabolic network of an individual organism [46].

### 3.3. Extraction of S-Layer Proteins from the Surface of L. brevis Strains

Following the protocol described in Čuljak et al. [14], *L. brevis* cells MB1, MB2, MB13 and MB20 were harvested after overnight incubation, washed in saline and suspended in 3 M guanidine hydrochloride (GHCl; Sigma-Aldrich, St. Louis, MO, USA) and incubated for 1 h at room temperature with shaking to obtain S-layer-depleted *L. brevis* strains (S−), or suspended in 5 M GHCl and incubated on ice for 2 h to obtain S-protein extracts. The S-protein extracts were separated from the cells by centrifugation at 4200 rpm for 10 min at 4 °C, and the cells were washed twice with saline to remove residual GHCl. Dialysis of S-protein extracts was performed using a 12–14 kDa Spectra/Por membrane (Spectrum Laboratories, Rancho Dominguez, CA, USA) in phosphate-buffered saline (PBS; pH = 7.4; Gibco, London, UK) at 4 °C on a magnetic stirrer. After dialysis, the samples were lyophilised in Christ Alpha 1–2 LDplus (Martin Christ, Osterode am Harz, Germany).

Untreated *L. brevis* cells (S+) were prepared for the experiments in the same way, except that distilled water was used instead of GHCl. To confirm the removal of the S-layer from the treated bacterial cells, the protein extracts were subjected to sodium dodecyl sulphate-polyacrylamide gel electrophoresis (SDS-PAGE) according to the protocol of Barukčić Jurina et al. [47]. The electrophoretic separation of the proteins was performed using a PowerPac HC™ (Biorad, Hercules, CA, USA) at a constant voltage of 150 V, along with the ProSieve QuadColor Protein Marker 4.6–315 kDa (Lonza, Morristown, NJ, USA).

### 3.4. Autoaggregation and Coaggregation Assay

Autoaggregation assay was performed as described in Leboš Pavunc et al. [48]. Previously prepared S-layer depleted (S−) and non-depleted (S+) *L. brevis* strains were washed twice with PBS (pH = 7.4). Washed bacterial cells were suspended in PBS to achieve an OD_620_ = 1 and autoaggregation was determined over a period of 5 h using the INFINITE F PLEX microplate reader (TECAN, Männedorf, Switzerland). The autoaggregation percentage is expressed as follows:

$$Autoaggregation\left(\%\right)=\left(1-\frac{A_{t}}{A_{0}}\right)\times 100$$

where A_t_ represents the absorbance at 5 h and A_0_ the absorbance at 0 h.

For the coaggregation assay, *E. coli* 3014 and *S.* Typhiumurium FP1 were prepared in the same way as *L. brevis* strains in autoaggregation assay. The percentage of coaggregation was calculated using the following equation:

$$Coaggregation\left(\%\right)=\frac{\frac{A_{x}+A_{y}}{2}-A\left(x+y\right)}{\frac{A_{x}+A_{y}}{2}}\times 100$$

where x and y represent each of the two bacterial strains in the experiment and x + y their mixture.

### 3.5. Competitive Exclusion Assay

The role of S-proteins of *L. brevis* strains MB1, MB2, MB13 and MB20 in the competitive exclusion of potentially pathogenic *E. coli* 3014 and *S.* Typhimurium FP1 was determined as described in Butorac et al. [10], with modifications. Untreated (S+) and GHCl-treated (S−) *L. brevis* strains were incubated at 37 °C for 1 h with a Caco-2 monolayer. After incubation, Caco-2 cells were washed with PBS (pH = 7.4) and 1 mL of suspension of potentially pathogenic bacteria was added to the wells and incubation was continued at 37 °C for 1 h. After incubation, the wells were washed 3 times with 1 mL PBS (pH = 7.4) to remove non-adherent bacterial cells and incubated for 10 min in 0.05% *v*/*v* Triton X-100 (AppliChem, Darmstadt, Germany) solution. The contents of each well were centrifuged at 13,000 rpm for 5 min. Finally, the cell precipitate was suspended in 1 mL PBS (pH = 7.4), and the total number of viable adherent *E. coli* 3014 and *S.* Typhimurium FP1 cells was determined using the spot-plate method on Rapid (Biorad, Hercules, CA, USA) and XLD (Biolife, Milan, Italy) agar plates, respectively.

### 3.6. The Effect of L. brevis and Their S-proteins on the Improvement of Epithelial Barrier Function In Vitro

#### 3.6.1. Cell Treatments

Caco-2 cell cultures at 80–90% confluent were washed three times with PBS and then incubated with either *L. brevis* strains (multiplicity of infection of 2) or their respective S-proteins (50 µg/mL) for 20 h. Caco-2 cells without any treatment were used as control. Experiments were also performed with simultaneous incubation of Caco-2 cells with either lipopolysaccharide (LPS) from *E. coli* (1 µg/mL) or TNF-α (10 ng/mL), or by their combined application, followed by the addition of *L. brevis* strains or S-proteins.

To analyse the capacity of *L. brevis* strains and their S-proteins to affect the expression of zymogen granule membrane protein 16 (ZG16), HT29 cells conditioned with methotrexate (MTX) and 5-fluorouracil (5FU) were also used. The HT29 cell sub clone was prepared by gradual adaption to the increasing concentrations of MTX and 5-FU (1 µM, 2 µM, 5 µM, and 10 µM) over 25–30 days. After conditioning, the cells were passaged and transferred into T25 flasks with medium without chemotherapeutics (DMEM/F12 + 10%FCS + L-Glu + Pen/Strep) and maintained in that medium. Next, HT29 cells were treated with *L. brevis* strains or S-proteins for 5 days. The effect was also monitored when the cells were simultaneously exposed to TNF-α (5 ng/mL) followed by treatment with *L. brevis* strains or S-proteins for 5 days.

#### 3.6.2. Immunofluorescent Microscopic Analysis of TJ Protein Expression

For immunolabelling of occludin (#CL488-27260) and ZO-1 (#CL488-21773), rabbit polyclonal antibodies conjugated to CoraLite^®^ Plus 488 (Proteintech, Rosemont, IL, USA) were used, while for JAM-A, a rabbit monoclonal antibody (clone EPR23244-12, Abcam, Cambridge, UK) and a secondary antibody conjugated with AlexaFluor 488 fluorochrome (Proteintech, Rosemont, IL, USA) were used. For immunolabelling of ZG16, a rabbit polyclonal antibody (#AB185483, Abcam, Cambridge, UK) and a secondary antibody conjugated with DyLight 488 fluorochrome (Abcam, Cambridge, UK) were used. The cell nuclei were stained with 4′,6-diamidino-2-phenylindole (DAPI; Sigma-Aldrich, St. Louis, MO, USA). For each antibody, a corresponding isotype control antibody was used in parallel.

After treatments, the cells were fixed by adding 70% *v*/*v* cold ethanol and permeabilised in a 0.1% solution of Triton-X 100 in PBS (pH = 7.4). After washing and blocking non-specific binding with 4% bovine serum albumin (BSA; Sigma-Aldrich, St. Louis, MO, USA), primary antibodies were added to the cells. If unlabelled antibodies were used, an additional step of incubating the cells with labelled secondary antibodies followed. Finally, the cells were spiked with 150 nM DAPI solution and then analysed using the EVOS FLc Cell Imager (Thermo Fisher Scientific, Waltham, MA, USA). For each sample, 6 images of cells at different positions in the well were captured and processed in ImageJ (https://imagej.nih.gov/ij/, accessed on 26 August 2024). The intensity of protein expression is expressed as the coefficient of mean fluorescence intensity (MFI) representing the ratio of the intensity of specific fluorescence and the isotypic control antibodies.

### 3.7. Flow Cytometric Measurement of Cytokines

The expression of IL-1β, IL-6, IL-8 and IL-10 cytokines was analysed after incubation of Caco-2 cells with *L. brevis* MB1, MB2, MB13 and MB20, or their S-proteins, under different treatments described in the previous chapter. Composite samples of media supernatant from individual wells (5 wells for each condition), stored at −80 °C, were used for analysis in duplicates after thawing. Samples were analysed using the cytometric bead array (CBA) method on an LSRII flow cytometer (Becton Dickinson, Franklin Lakes, NJ, USA). Frozen cell media were thawed and prepared for flow cytometer analysis according to the CBA kit LEGENDplex (Biolegend, San Diego, CA, USA) manufacturer’s instructions for selected cytokines. The obtained fluorescence data were analysed using GainData^®^ (arigo’s ELISA Calculator, available at the link https://www.arigobio.com/ELISA-calculator, accessed on 9 September 2024).

### 3.8. Ability of L. brevis Strains to Adhere to Recombinant Human ZG16 Protein

In order to test the ability of selected strains to adhere to the ZG16 protein, the ZG16 protein (#ab276544, Abcam, Cambridge, UK) or the I-Block reagent (control; Thermo Fisher Scientific, Waltham, MA, USA), both at a concentration of 8 µg/mL, were added to a 96-well microtitre plate and incubated for 4 h. After incubation, the contents of the wells were removed and 1% BSA solution was added, followed by overnight incubation at 4 °C. To achieve the desired number of bacteria per well (50,000, 25,000 and 12,500 bacteria), the corresponding volumes of bacterial suspension and medium were added to the wells, followed by overnight incubation (16 h) at 37 °C and 5% CO_2_. After incubation, the wells were washed three times with PBS preheated to 37 °C followed with 10 mM dithiothreitol (Thermo Fisher Scientific, Waltham, MA, USA) in PBS treatment for 15 min to detach adhered bacteria. The contents of the wells were transferred to sterile tubes and the wells were additionally washed with PBS or 1% BSA in PBS. Pooled bacterial suspensions were centrifuged at 3220× *g* for 15 min. The bacteria in the pellet were suspended in 50 μL of PBS and inoculated in duplicate of 25 μL on previously prepared MRS agar plates. After 48 h of anaerobic incubation at 37 °C, the grown colonies were counted.

### 3.9. Statistical Analysis

The results are expressed as the mean of three independent values ± standard deviation. Statistical significance was determined using one-way analysis of variance (ANOVA). Pairwise differences between group means were determined using Tukey’s honestly significant difference test for pairwise comparisons after analysis of variance (https://www.statskingdom.com/index.html, accessed on 2 September 2024) [49]. Differences between samples were considered significant at *p* < 0.05. Images were created using GraphPad Prism v.9.4.1 (GraphPad Software, La Jolla, CA, USA).

## 4. Conclusions

The role of S-proteins in the autoaggregation, coaggregation, and competitive exclusion of *S.* Typhimurium FP1 of all examined strains as well as in the coaggregation with *E. coli* 3014 of strains MB1 and MB2 and competitive exclusion against *E. coli* 3014 of strains MB13 and MB20 were demonstrated. In vitro experiments on human cell cultures demonstrated the beneficial effect on intestinal cell barrier function as evident from the capacity of increase in the expression of JAM-A, occludin and ZG16 proteins, which are required for the maintenance of intestinal integrity. Furthermore, the immunomodulatory effect of the *L. brevis* strains and their S-proteins was detected as a decrease in the production of the pro-inflammatory cytokines IL-6, IL-8 and IL-1β and an increase in the production of the anti-inflammatory cytokine IL-10. In addition, all tested *L. brevis* strains were found to bind to the ZG16 protein, with strain MB20 exhibiting the highest affinity and adhesion capacity for the human ZG16 protein. Finally, to conclude, the study supports in vitro evidence for various aspects of the beneficial activities of *L. brevis* strains such as aggregation, competitive exclusion, effect on tight junction proteins and cytokine expression, in the context of the involvement of S-layer proteins in these processes. This contributes to the characterisation of the probiotic mechanisms of action of S-layer producing *L. brevis* strains and stimulates further research important to their adaptation in the intestinal microenvironment and thus gut functionality.

## Figures and Tables

**Table 1:** Autoaggregation ability (%) of untreated (S+) and GHCl-treated (S−) *Levilactobactobacillus brevis* MB1, MB2, MB13 and MB20 strains.

	Autoaggregation %	
*L. brevis*	Untreated (S+)	GHCl-Treated (S−)
MB1	73.57 ± 5.91 ^a^	26.44 ± 4.57 ^b^
MB2	66.04 ± 8.33 ^a^	42.53 ± 7.18 ^b^
MB13	77.15 ± 4.89 ^a^	41.51 ± 12.57 ^b^
MB20	69.65 ± 5.27 ^a^	21.92 ± 3.26 ^b^

Results are reported as mean values ± standard deviation. Different letters in superscript indicate a statistically significant difference (*p* < 0.05) between S+ and S− of the same strain.

**Table 2:** Coaggregation ability (%) of untreated (S+) and GHCl-treated (S−) *Levilactobacillus brevis* MB1, MB2, MB13 and MB20 strains with *Escherichia coli* 3014 and *Salmonella enterica* serovar Typhimurium FP1.

*L. brevis*	Coaggregation %			
	*S.* Typhimurium FP1		*E. coli* 3014	
	Untreated (S+)	GHCl-Treated (S−)	Untreated (S+)	GHCl-Treated (S−)
MB1	47.07 ± 8.05 ^a^	19.62 ± 2.71 ^b^	41.54 ± 2.96 ^a^	28.02 ± 3.74 ^b^
MB2	46.73 ± 8.04 ^a^	16.97 ± 0.96 ^b^	32.41 ± 4.35 ^a^	18.59 ± 2.69 ^b^
MB13	26.36 ± 1.14 ^a^	16.35 ± 0.94 ^b^	26.34 ± 4.07 ^a^	36.72 ± 5.06 ^a^
MB20	34.63 ± 3.17 ^a^	22.20 ± 1.64 ^b^	35.12 ± 8.42 ^a^	23.55 ± 4.43 ^a^

Results are reported as mean values ± standard deviation. Different letters in superscript indicate a statistically significant difference (*p* < 0.05) between S+ and S− of the same strain.

## Data Availability

The original contributions presented in this study are included in the article and Supplementary Materials. Further inquiries can be directed to the corresponding author.

## Supplementary Materials

The following supporting information can be downloaded at: www.mdpi.com/article/10.3390/ijms26062425/s1.
