# Supplementary material for: In Vitro Analysis of Probiotic Properties Related to the Adaptation of Levilactobacillus brevis to Intestinal Microenvironment and Involvement of S-Layer Proteins
Source: Int J Mol Sci. 2025 Mar 7;26(6):2425. doi: 10.3390/ijms26062425 (PMC11942123; doi:10.3390/ijms26062425)
Supplement: Supplementary file 1 [file ijms-26-02425-s001.zip › Figure S2.pdf]

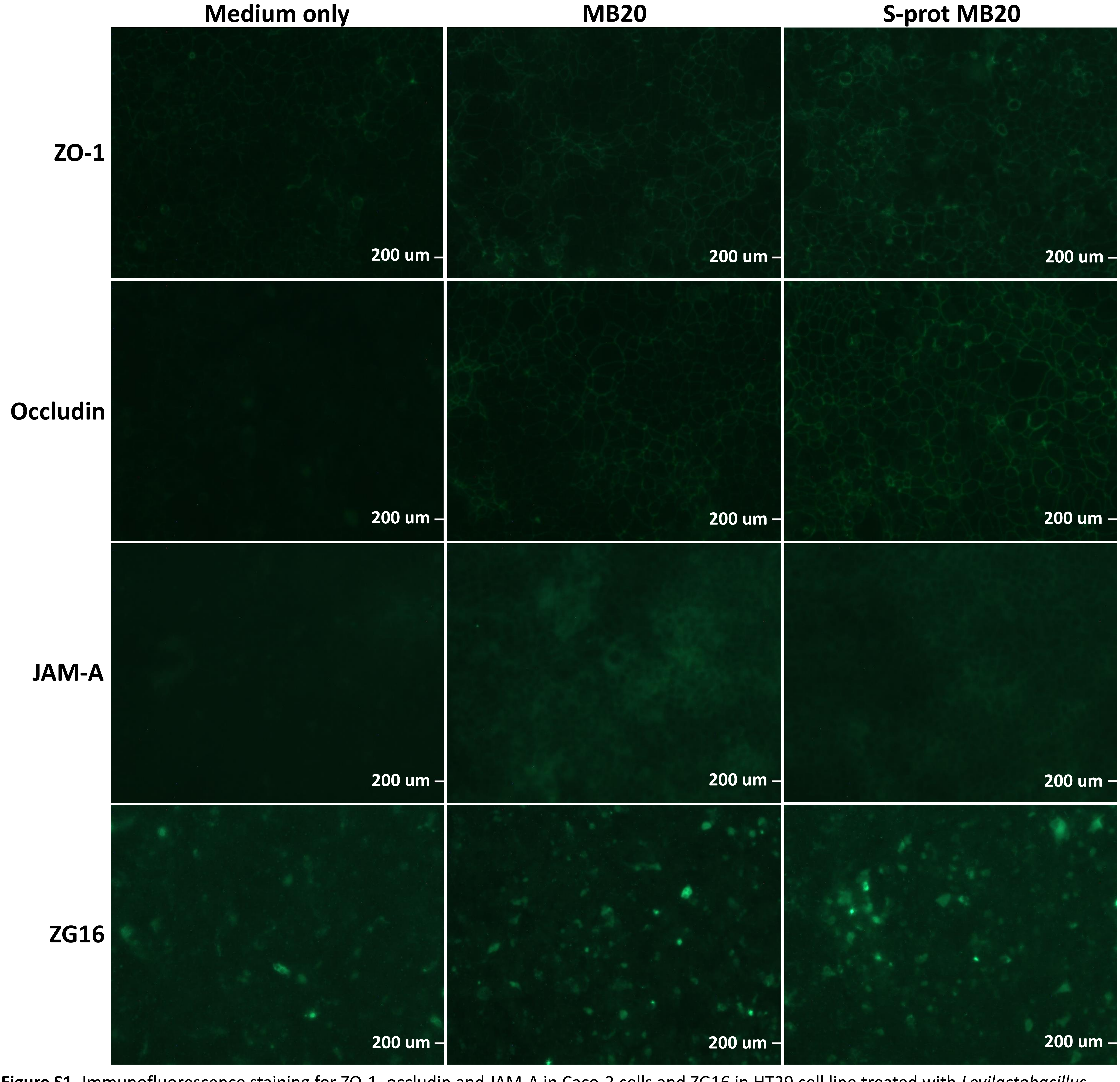

Figure S1. Immunofluorescence staining for ZO-1, occludin and JAM-A in Caco-2 cells and ZG16 in HT29 cell line treated with *Levilactobacillus brevis* MB20 (MB20) and their S-proteins (S-prot MB20) (control - medium only). Scale bar = 200 um.
