# Supplementary material for: In Vitro Analysis of Probiotic Properties Related to the Adaptation of Levilactobacillus brevis to Intestinal Microenvironment and Involvement of S-Layer Proteins
Source: Int J Mol Sci. 2025 Mar 7;26(6):2425. doi: 10.3390/ijms26062425 (PMC11942123; doi:10.3390/ijms26062425)
Supplement: Supplementary file 1 [file ijms-26-02425-s001.zip › Table S1.pdf]

Table S1. Comparative genome analysis of *Levilactobacillus brevis* strains MB1, MB2, MB13 and MB20 obtained using RAST server

**Genome data**	**LAB strain**			
	**MB1**	**MB2**	**MB13**	**MB20**
**size (bp)**	2 452 512	2 462 442	2 443 961	2 462 783
**GC content (%)**	45.9	45.9	46.0	45.9
**N50**	67352	67325	67352	67352
**L50**	12	12	12	12
**number of contigs (with PEGs)**	67	68	66	69
**number of subsystems**	214	216	214	216
**number of coding sequences**	2490	2500	2476	2507
**number of RNAs**	45	62	45	52

N50 – the sequence length of the shortest contig at 50 % of the total assembly length

L50 – count of smallest number of contigs whose length sum makes up half of genome size

PEG – protein encoding genes
