# Supplementary figures and images for: In Vitro Analysis of Probiotic Properties Related to the Adaptation of Levilactobacillus brevis to Intestinal Microenvironment and Involvement of S-Layer Proteins
Source: Int J Mol Sci. 2025 Mar 7;26(6):2425. doi: 10.3390/ijms26062425 (PMC11942123; doi:10.3390/ijms26062425)

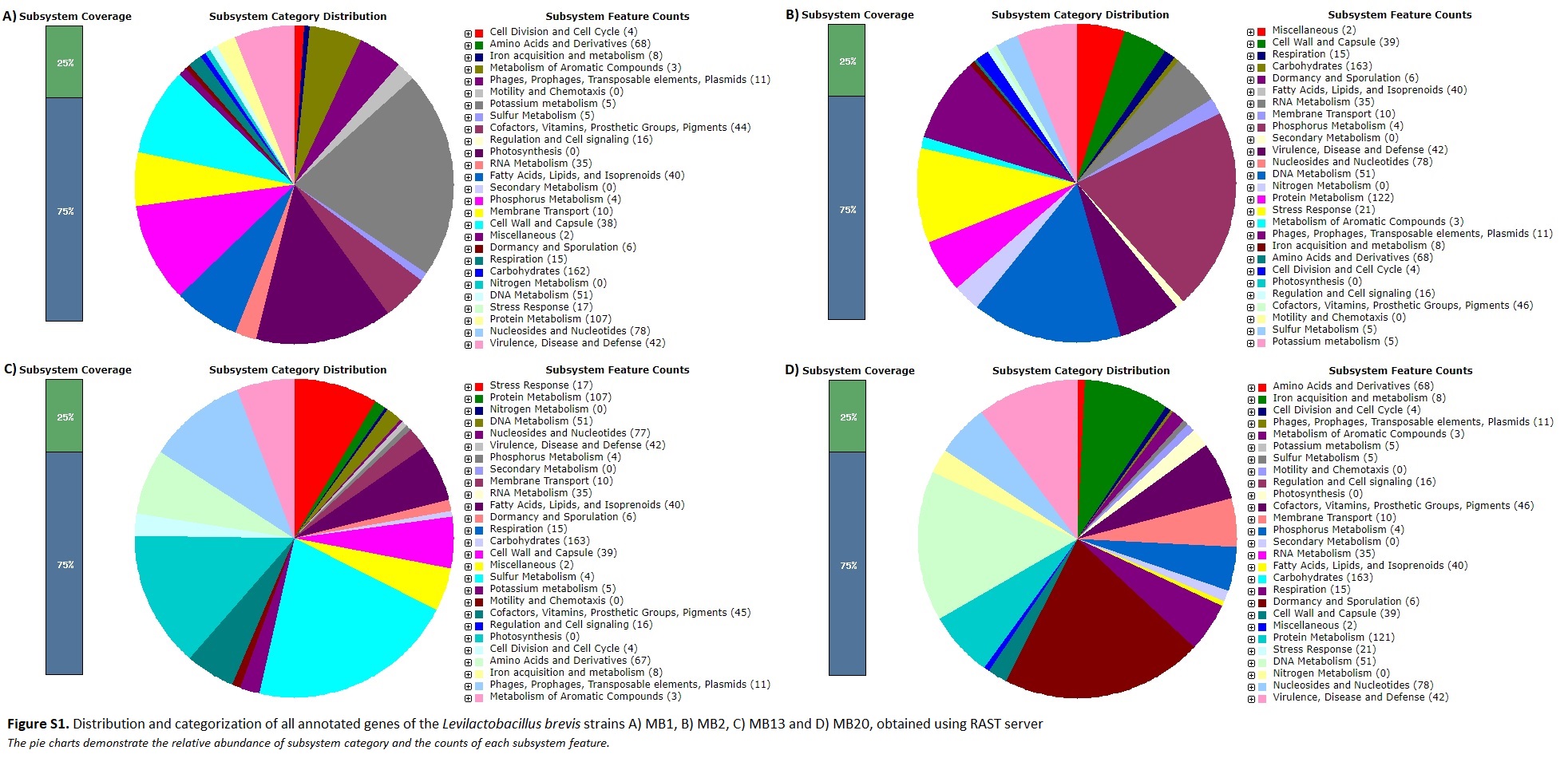

Supplement: Supplementary file 1 [file ijms-26-02425-s001.zip › Figure S1.jpg]
